# Impact of Prolonged Light‐Emitting Diode Exposure on Serotonin Gene Expression and Gut Microbiota in a Rat Model

**DOI:** 10.1002/alz70862_110067

**Published:** 2025-12-23

**Authors:** Adejoke Olukayode Olukayode Obajuluwa, James Chrystopher Lech, Tiwalola Madoc Obajuluwa, Pius Abimbola Okiki

**Affiliations:** ^1^ Afe Babalola University, Ado ‐Ekiti, Ekiti State Nigeria; ^2^ ABUAD Biotechnology Centre, Ado ‐Ekiti, Ekiti Nigeria; ^3^ University Amsterdam Medical Centre, Amsterdam Netherlands; ^4^ Afe Babalola University, Ado‐Ekiti, Ekiti Nigeria; ^5^ Afe Babalola University, Ado Nigeria

## Abstract

**Background:**

Excessive nighttime exposure to light‐emitting diode (LED) has been implicated in gut‐brain axis dysfunction, leading to disrupted serotonin levels that regulate essential functions such as mood, sleep, appetite, and cognition. Hence, this study investigated the effects of prolonged LED exposure on weight, gut microbiota and serotonin gene expression in male Wistar rats.

**Method:**

Twenty male Wistar rats, each weighing between 200g and 220g, were divided into two groups (*n* = 10 each): (i) a control group maintained on a natural 12‐hour light/dark cycle and (ii) an experimental group exposed to 24‐hour LED light for five weeks. Brain serotonin (5‐HT) mRNA expression was analyzed using conventional and real‐time PCR, with electrophoresis on 1% agarose gels and visualization via SafeView staining. Gut microbiota composition was assessed from colons through next‐generation sequencing (NGS) on the PacBio Sequel IIe platform, and taxonomic data were analyzed using QIIME2. Statistical analyses were performed using GraphPad 9 with significance set at *p* ≤0.05.

**Result:**

We observed a significant increase in weight gain (*p* <0.01) and a reduction in serotonin gene expression in the LED‐exposed group compared to controls (*p* <0.001). Taxonomic analysis revealed substantial alterations in colon microbiota composition, including a decrease in Actinobacteriota and Proteobacteria in the LED group (*p* <0.0001), alongside an increased relative abundance of Firmicutes (*p* <0.01) compared to the control group. Also, bacterial diversity was significantly higher in the LED group (28.09%) compared to the control group (23.96%).

**Conclusion:**

These findings suggest that prolonged exposure to LED light disrupts serotonin gene expression and alters gut microbiota composition, potentially impairing gut‐brain axis function. To mitigate these effects, limiting excessive nighttime LED exposure is recommended.

